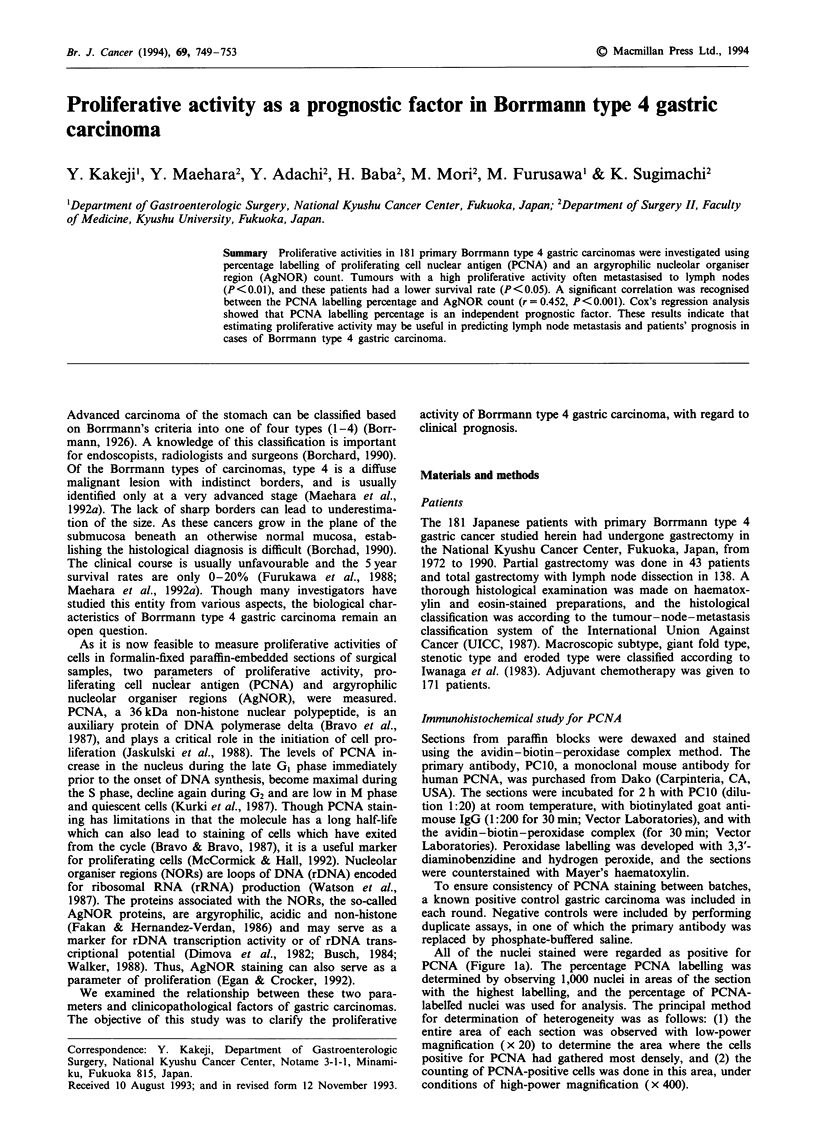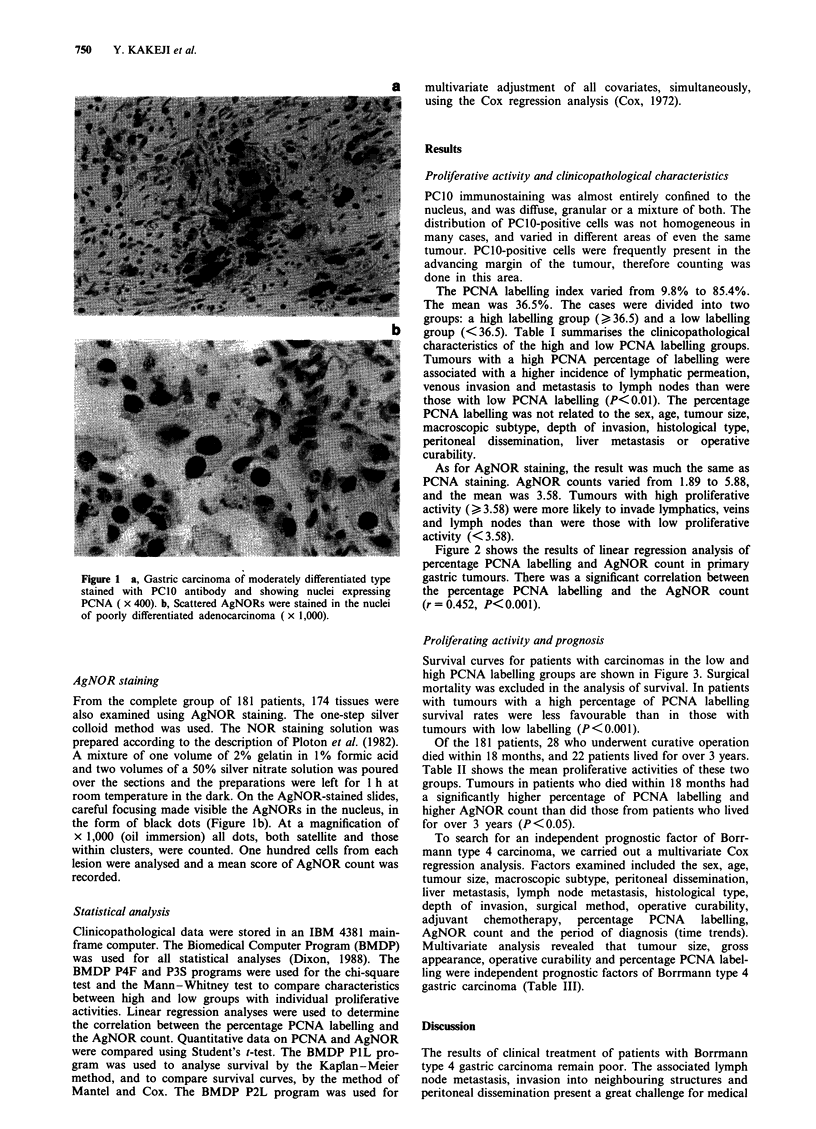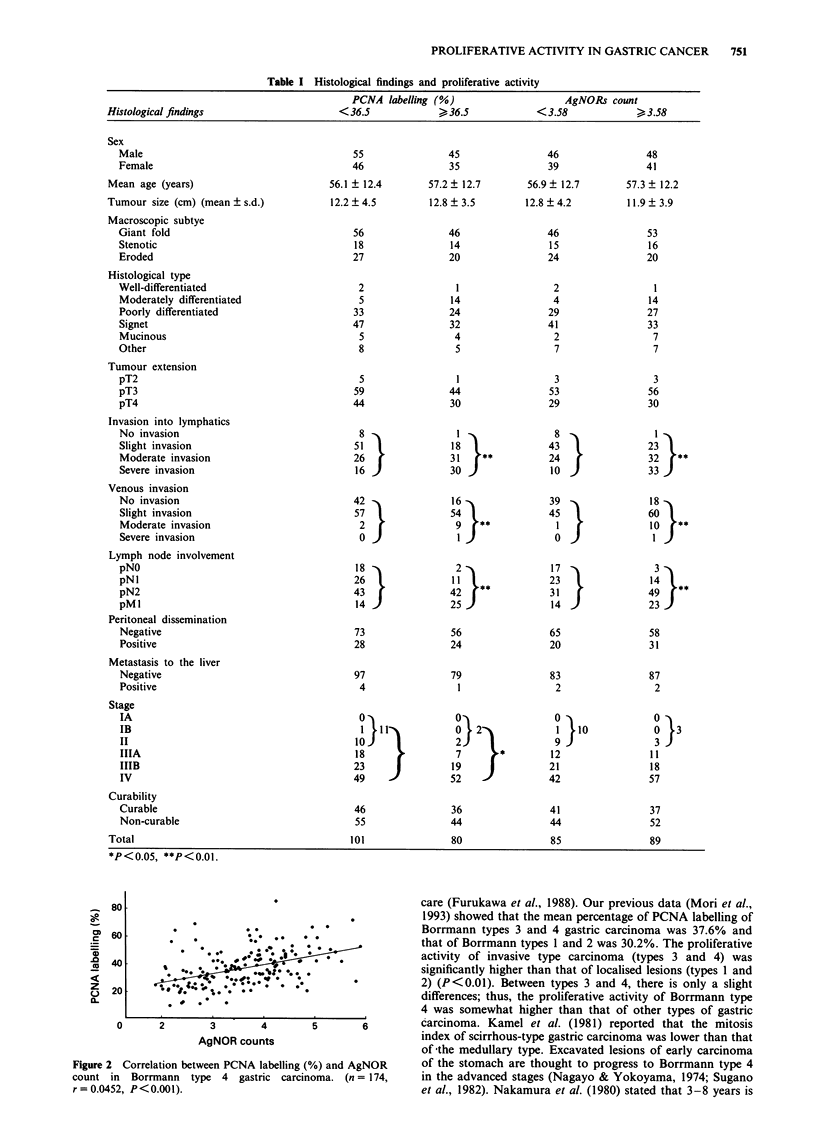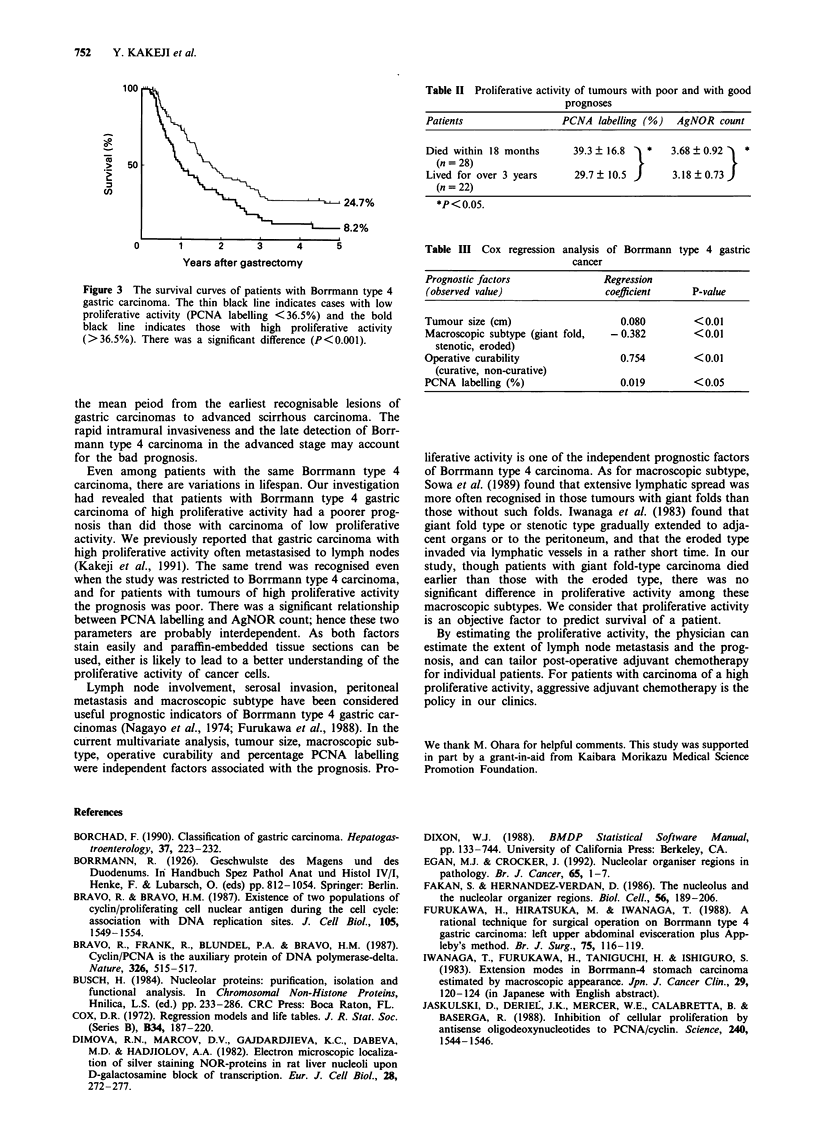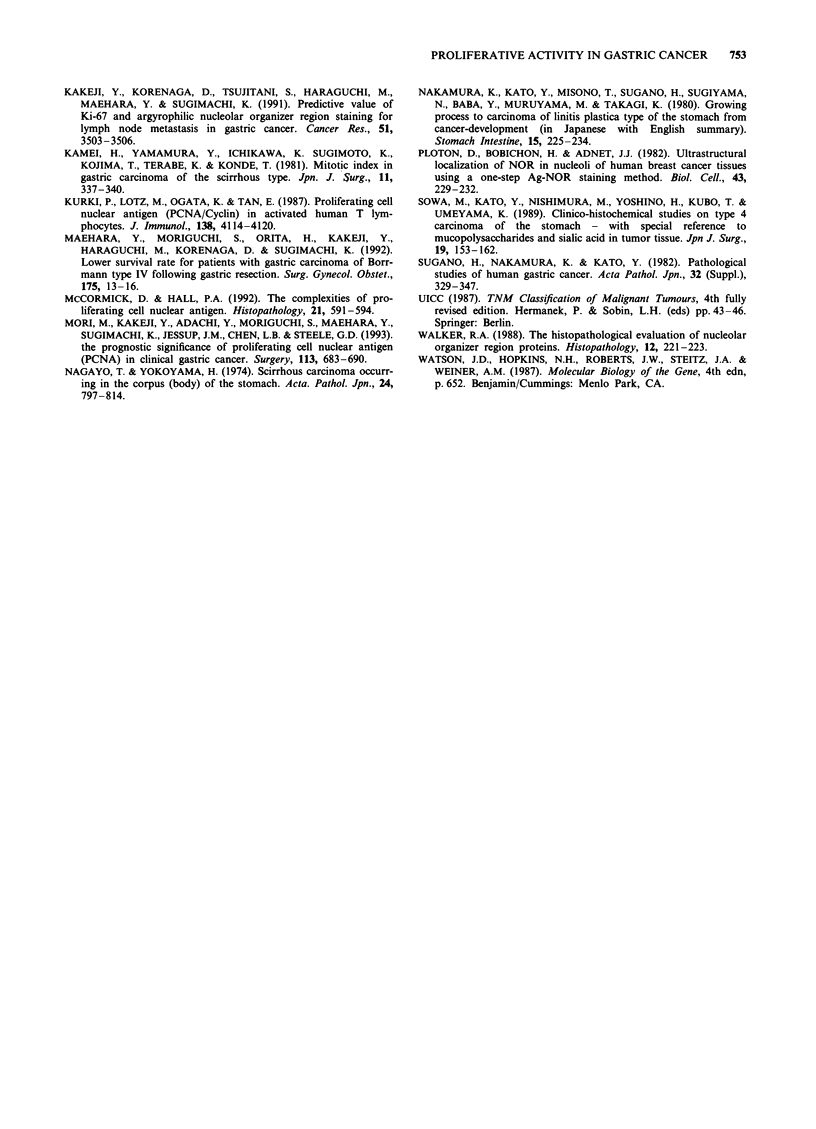# Proliferative activity as a prognostic factor in Borrmann type 4 gastric carcinoma.

**DOI:** 10.1038/bjc.1994.141

**Published:** 1994-04

**Authors:** Y. Kakeji, Y. Maehara, Y. Adachi, H. Baba, M. Mori, M. Furusawa, K. Sugimachi

**Affiliations:** Department of Gastroenterologic Surgery, National Kyushu Cancer Center, Fukuoka, Japan.

## Abstract

**Images:**


Br. J. Cancer (1994), 69, 749-753 © Macmillan Press Ltd., 1994

### Proliferative activity as a prognostic factor in Borrmann type 4 gastric carcinoma

Y. Kakeji[1], Y. Maehara[2], Y. Adachi[2], H. Baba[2], M. Mori[2], M. Furusawa[1] & K. Sugimachi[2]

[1]*Department of Gastroenterologic Surgery, National Kyushu Cancer Center, Fukuoka, Japan;* [2]*Department of Surgery II, Faculty of Medicine, Kyushu University, Fukuoka, Japan.*

**Summary** Proliferative activities in 181 primary Borrmann type 4 gastric carcinomas were investigated using percentage labelling of proliferating cell nuclear antigen (PCNA) and an argyrophilic nucleolar organiser region (AgNOR) count. Tumours with a high proliferative activity often metastasised to lymph nodes ($P<0.01$), and these patients had a lower survival rate ($P<0.05$). A significant correlation was recognised between the PCNA labelling percentage and AgNOR count ($r = 0.452$, $P<0.001$). Cox's regression analysis showed that PCNA labelling percentage is an independent prognostic factor. These results indicate that estimating proliferative activity may be useful in predicting lymph node metastasis and patients' prognosis in cases of Borrmann type 4 gastric carcinoma.

Advanced carcinoma of the stomach can be classified based on Borrmann's criteria into one of four types (1-4) (Borrmann, 1926). A knowledge of this classification is important for endoscopists, radiologists and surgeons (Borchard, 1990). Of the Borrmann types of carcinomas, type 4 is a diffuse malignant lesion with indistinct borders, and is usually identified only at a very advanced stage (Maehara *et al.*, 1992*a*). The lack of sharp borders can lead to underestimation of the size. As these cancers grow in the plane of the submucosa beneath an otherwise normal mucosa, establishing the histological diagnosis is difficult (Borchad, 1990). The clinical course is usually unfavourable and the 5 year survival rates are only 0-20% (Furukawa *et al.*, 1988; Maehara *et al.*, 1992*a*). Though many investigators have studied this entity from various aspects, the biological characteristics of Borrmann type 4 gastric carcinoma remain an open question.

As it is now feasible to measure proliferative activities of cells in formalin-fixed paraffin-embedded sections of surgical samples, two parameters of proliferative activity, proliferating cell nuclear antigen (PCNA) and argyrophilic nucleolar organiser regions (AgNOR), were measured. PCNA, a 36 kDa non-histone nuclear polypeptide, is an auxiliary protein of DNA polymerase delta (Bravo *et al.*, 1987), and plays a critical role in the initiation of cell proliferation (Jaskulski *et al.*, 1988). The levels of PCNA increase in the nucleus during the late $G_1$ phase immediately prior to the onset of DNA synthesis, become maximal during the S phase, decline again during $G_2$ and are low in M phase and quiescent cells (Kurki *et al.*, 1987). Though PCNA staining has limitations in that the molecule has a long half-life which can also lead to staining of cells which have exited from the cycle (Bravo & Bravo, 1987), it is a useful marker for proliferating cells (McCormick & Hall, 1992). Nucleolar organiser regions (NORs) are loops of DNA (rDNA) encoded for ribosomal RNA (rRNA) production (Watson *et al.*, 1987). The proteins associated with the NORs, the so-called AgNOR proteins, are argyrophilic, acidic and non-histone (Fakan & Hernandez-Verdan, 1986) and may serve as a marker for rDNA transcription activity or of rDNA transcriptional potential (Dimova *et al.*, 1982; Busch, 1984; Walker, 1988). Thus, AgNOR staining can also serve as a parameter of proliferation (Egan & Crocker, 1992).

We examined the relationship between these two parameters and clinicopathological factors of gastric carcinomas. The objective of this study was to clarify the proliferative activity of Borrmann type 4 gastric carcinoma, with regard to clinical prognosis.

Correspondence: Y. Kakeji, Department of Gastroenterologic Surgery, National Kyushu Cancer Center, Notame 3-1-1, Minami-ku, Fukuoka 815, Japan.
Received 10 August 1993; and in revised form 12 November 1993.

### Materials and methods

### *Patients*

The 181 Japanese patients with primary Borrmann type 4 gastric cancer studied herein had undergone gastrectomy in the National Kyushu Cancer Center, Fukuoka, Japan, from 1972 to 1990. Partial gastrectomy was done in 43 patients and total gastrectomy with lymph node dissection in 138. A thorough histological examination was made on haematoxylin and eosin-stained preparations, and the histological classification was according to the tumour-node-metastasis classification system of the International Union Against Cancer (UICC, 1987). Macroscopic subtype, giant fold type, stenotic type and eroded type were classified according to Iwanaga *et al.* (1983). Adjuvant chemotherapy was given to 171 patients.

### *Immunohistochemical study for PCNA*

Sections from paraffin blocks were dewaxed and stained using the avidin-biotin-peroxidase complex method. The primary antibody, PC10, a monoclonal mouse antibody for human PCNA, was purchased from Dako (Carpinteria, CA, USA). The sections were incubated for 2 h with PC10 (dilution 1:20) at room temperature, with biotinylated goat anti-mouse IgG (1:200 for 30 min; Vector Laboratories), and with the avidin-biotin-peroxidase complex (for 30 min; Vector Laboratories). Peroxidase labelling was developed with 3,3'-diaminobenzidine and hydrogen peroxide, and the sections were counterstained with Mayer's haematoxylin.

To ensure consistency of PCNA staining between batches, a known positive control gastric carcinoma was included in each round. Negative controls were included by performing duplicate assays, in one of which the primary antibody was replaced by phosphate-buffered saline.

All of the nuclei stained were regarded as positive for PCNA (Figure 1a). The percentage PCNA labelling was determined by observing 1,000 nuclei in areas of the section with the highest labelling, and the percentage of PCNA-labelled nuclei was used for analysis. The principal method for determination of heterogeneity was as follows: (1) the entire area of each section was observed with low-power magnification ($\times$ 20) to determine the area where the cells positive for PCNA had gathered most densely, and (2) the counting of PCNA-positive cells was done in this area, under conditions of high-power magnification ($\times$ 400).

**Figure 1** **a**, Gastric carcinoma of moderately differentiated type stained with PC10 antibody and showing nuclei expressing PCNA ( × 400). **b**, Scattered AgNORs were stained in the nuclei of poorly differentiated adenocarcinoma ( × 1,000).

*AgNOR staining*

From the complete group of 181 patients, 174 tissues were also examined using AgNOR staining. The one-step silver colloid method was used. The NOR staining solution was prepared according to the description of Ploton *et al.* (1982). A mixture of one volume of 2% gelatin in 1% formic acid and two volumes of a 50% silver nitrate solution was poured over the sections and the preparations were left for 1 h at room temperature in the dark. On the AgNOR-stained slides, careful focusing made visible the AgNORs in the nucleus, in the form of black dots (Figure 1b). At a magnification of × 1,000 (oil immersion) all dots, both satellite and those within clusters, were counted. One hundred cells from each lesion were analysed and a mean score of AgNOR count was recorded.

*Statistical analysis*

Clinicopathological data were stored in an IBM 4381 mainframe computer. The Biomedical Computer Program (BMDP) was used for all statistical analyses (Dixon, 1988). The BMDP P4F and P3S programs were used for the chi-square test and the Mann–Whitney test to compare characteristics between high and low groups with individual proliferative activities. Linear regression analyses were used to determine the correlation between the percentage PCNA labelling and the AgNOR count. Quantitative data on PCNA and AgNOR were compared using Student's *t*-test. The BMDP P1L program was used to analyse survival by the Kaplan–Meier method, and to compare survival curves, by the method of Mantel and Cox. The BMDP P2L program was used for multivariate adjustment of all covariates, simultaneously, using the Cox regression analysis (Cox, 1972).

### Results

*Proliferative activity and clinicopathological characteristics*

PC10 immunostaining was almost entirely confined to the nucleus, and was diffuse, granular or a mixture of both. The distribution of PC10-positive cells was not homogeneous in many cases, and varied in different areas of even the same tumour. PC10-positive cells were frequently present in the advancing margin of the tumour, therefore counting was done in this area.

The PCNA labelling index varied from 9.8% to 85.4%. The mean was 36.5%. The cases were divided into two groups: a high labelling group ($\geqslant 36.5$) and a low labelling group ($< 36.5$). Table I summarises the clinicopathological characteristics of the high and low PCNA labelling groups. Tumours with a high PCNA percentage of labelling were associated with a higher incidence of lymphatic permeation, venous invasion and metastasis to lymph nodes than were those with low PCNA labelling ($P < 0.01$). The percentage PCNA labelling was not related to the sex, age, tumour size, macroscopic subtype, depth of invasion, histological type, peritoneal dissemination, liver metastasis or operative curability.

As for AgNOR staining, the result was much the same as PCNA staining. AgNOR counts varied from 1.89 to 5.88, and the mean was 3.58. Tumours with high proliferative activity ($\geqslant 3.58$) were more likely to invade lymphatics, veins and lymph nodes than were those with low proliferative activity ($< 3.58$).

Figure 2 shows the results of linear regression analysis of percentage PCNA labelling and AgNOR count in primary gastric tumours. There was a significant correlation between the percentage PCNA labelling and the AgNOR count ($r = 0.452$, $P < 0.001$).

*Proliferating activity and prognosis*

Survival curves for patients with carcinomas in the low and high PCNA labelling groups are shown in Figure 3. Surgical mortality was excluded in the analysis of survival. In patients with tumours with a high percentage of PCNA labelling survival rates were less favourable than in those with tumours with low labelling ($P < 0.001$).

Of the 181 patients, 28 who underwent curative operation died within 18 months, and 22 patients lived for over 3 years. Table II shows the mean proliferative activities of these two groups. Tumours in patients who died within 18 months had a significantly higher percentage of PCNA labelling and higher AgNOR count than did those from patients who lived for over 3 years ($P < 0.05$).

To search for an independent prognostic factor of Borrmann type 4 carcinoma, we carried out a multivariate Cox regression analysis. Factors examined included the sex, age, tumour size, macroscopic subtype, peritoneal dissemination, liver metastasis, lymph node metastasis, histological type, depth of invasion, surgical method, operative curability, adjuvant chemotherapy, percentage PCNA labelling, AgNOR count and the period of diagnosis (time trends). Multivariate analysis revealed that tumour size, gross appearance, operative curability and percentage PCNA labelling were independent prognostic factors of Borrmann type 4 gastric carcinoma (Table III).

### Discussion

The results of clinical treatment of patients with Borrmann type 4 gastric carcinoma remain poor. The associated lymph node metastasis, invasion into neighbouring structures and peritoneal dissemination present a great challenge for medical

**Table I** Histological findings and proliferative activity

| *Histological findings* | *PCNA labelling (%)* <*36.5* | ≥*36.5* | *AgNORs count* <*3.58* | ≥*3.58* |
|---|---|---|---|---|
| Sex | | | | |
| Male | 55 | 45 | 46 | 48 |
| Female | 46 | 35 | 39 | 41 |
| Mean age (years) | 56.1 ± 12.4 | 57.2 ± 12.7 | 56.9 ± 12.7 | 57.3 ± 12.2 |
| Tumour size (cm) (mean ± s.d.) | 12.2 ± 4.5 | 12.8 ± 3.5 | 12.8 ± 4.2 | 11.9 ± 3.9 |
| Macroscopic subtye | | | | |
| Giant fold | 56 | 46 | 46 | 53 |
| Stenotic | 18 | 14 | 15 | 16 |
| Eroded | 27 | 20 | 24 | 20 |
| Histological type | | | | |
| Well-differentiated | 2 | 1 | 2 | 1 |
| Moderately differentiated | 5 | 14 | 4 | 14 |
| Poorly differentiated | 33 | 24 | 29 | 27 |
| Signet | 47 | 32 | 41 | 33 |
| Mucinous | 5 | 4 | 2 | 7 |
| Other | 8 | 5 | 7 | 7 |
| Tumour extension | | | | |
| pT2 | 5 | 1 | 3 | 3 |
| pT3 | 59 | 44 | 53 | 56 |
| pT4 | 44 | 30 | 29 | 30 |
| Invasion into lymphatics | | | | |
| No invasion | 8 | 1 | 8 | 1 |
| Slight invasion | 51 | 18 | 43 | 23 |
| Moderate invasion | 26 | 31 ** | 24 | 32 ** |
| Severe invasion | 16 | 30 | 10 | 33 |
| Venous invasion | | | | |
| No invasion | 42 | 16 | 39 | 18 |
| Slight invasion | 57 | 54 | 45 | 60 |
| Moderate invasion | 2 | 9 ** | 1 | 10 ** |
| Severe invasion | 0 | 1 | 0 | 1 |
| Lymph node involvement | | | | |
| pN0 | 18 | 2 | 17 | 3 |
| pN1 | 26 | 11 | 23 | 14 |
| pN2 | 43 | 42 ** | 31 | 49 ** |
| pM1 | 14 | 25 | 14 | 23 |
| Peritoneal dissemination | | | | |
| Negative | 73 | 56 | 65 | 58 |
| Positive | 28 | 24 | 20 | 31 |
| Metastasis to the liver | | | | |
| Negative | 97 | 79 | 83 | 87 |
| Positive | 4 | 1 | 2 | 2 |
| Stage | | | | |
| IA | 0 | 0 | 0 | 0 |
| IB | 1 } 11 | 0 } 2 | 1 } 10 | 0 } 3 |
| II | 10 | 2 | 9 | 3 |
| IIIA | 18 | 7 * | 12 | 11 |
| IIIB | 23 | 19 | 21 | 18 |
| IV | 49 | 52 | 42 | 57 |
| Curability | | | | |
| Curable | 46 | 36 | 41 | 37 |
| Non-curable | 55 | 44 | 44 | 52 |
| Total | 101 | 80 | 85 | 89 |

$^{*}P<0.05$, $^{**}P<0.01$.

**Figure 2** Correlation between PCNA labelling (%) and AgNOR count in Borrmann type 4 gastric carcinoma. ($n = 174$, $r = 0.0452$, $P<0.001$).

care (Furukawa *et al.*, 1988). Our previous data (Mori *et al.*, 1993) showed that the mean percentage of PCNA labelling of Borrmann types 3 and 4 gastric carcinoma was 37.6% and that of Borrmann types 1 and 2 was 30.2%. The proliferative activity of invasive type carcinoma (types 3 and 4) was significantly higher than that of localised lesions (types 1 and 2) ($P<0.01$). Between types 3 and 4, there is only a slight differences; thus, the proliferative activity of Borrmann type 4 was somewhat higher than that of other types of gastric carcinoma. Kamel *et al.* (1981) reported that the mitosis index of scirrhous-type gastric carcinoma was lower than that of the medullary type. Excavated lesions of early carcinoma of the stomach are thought to progress to Borrmann type 4 in the advanced stages (Nagayo & Yokoyama, 1974; Sugano *et al.*, 1982). Nakamura *et al.* (1980) stated that 3–8 years is

Figure 3 The survival curves of patients with Borrmann type 4 gastric carcinoma. The thin black line indicates cases with low proliferative activity (PCNA labelling <36.5%) and the bold black line indicates those with high proliferative activity (>36.5%). There was a significant difference ($P<0.001$).

**Table II** Proliferative activity of tumours with poor and with good prognoses

| *Patients* | *PCNA labelling (%)* | *AgNOR count* |
|---|---|---|
| Died within 18 months ($n = 28$) | 39.3 ± 16.8 } * | 3.68 ± 0.92 } * |
| Lived for over 3 years ($n = 22$) | 29.7 ± 10.5 | 3.18 ± 0.73 |

$*P<0.05$.

**Table III** Cox regression analysis of Borrmann type 4 gastric cancer

| *Prognostic factors (observed value)* | *Regression coefficient* | P-*value* |
|---|---|---|
| Tumour size (cm) | 0.080 | <0.01 |
| Macroscopic subtype (giant fold, stenotic, eroded) | − 0.382 | <0.01 |
| Operative curability (curative, non-curative) | 0.754 | <0.01 |
| PCNA labelling (%) | 0.019 | <0.05 |

the mean peiod from the earliest recognisable lesions of gastric carcinomas to advanced scirrhous carcinoma. The rapid intramural invasiveness and the late detection of Borrmann type 4 carcinoma in the advanced stage may account for the bad prognosis.

Even among patients with the same Borrmann type 4 carcinoma, there are variations in lifespan. Our investigation had revealed that patients with Borrmann type 4 gastric carcinoma of high proliferative activity had a poorer prognosis than did those with carcinoma of low proliferative activity. We previously reported that gastric carcinoma with high proliferative activity often metastasised to lymph nodes (Kakeji *et al.*, 1991). The same trend was recognised even when the study was restricted to Borrmann type 4 carcinoma, and for patients with tumours of high proliferative activity the prognosis was poor. There was a significant relationship between PCNA labelling and AgNOR count; hence these two parameters are probably interdependent. As both factors stain easily and paraffin-embedded tissue sections can be used, either is likely to lead to a better understanding of the proliferative activity of cancer cells.

Lymph node involvement, serosal invasion, peritoneal metastasis and macroscopic subtype have been considered useful prognostic indicators of Borrmann type 4 gastric carcinomas (Nagayo *et al.*, 1974; Furukawa *et al.*, 1988). In the current multivariate analysis, tumour size, macroscopic subtype, operative curability and percentage PCNA labelling were independent factors associated with the prognosis. Proliferative activity is one of the independent prognostic factors of Borrmann type 4 carcinoma. As for macroscopic subtype, Sowa *et al.* (1989) found that extensive lymphatic spread was more often recognised in those tumours with giant folds than those without such folds. Iwanaga *et al.* (1983) found that giant fold type or stenotic type gradually extended to adjacent organs or to the peritoneum, and that the eroded type invaded via lymphatic vessels in a rather short time. In our study, though patients with giant fold-type carcinoma died earlier than those with the eroded type, there was no significant difference in proliferative activity among these macroscopic subtypes. We consider that proliferative activity is an objective factor to predict survival of a patient.

By estimating the proliferative activity, the physician can estimate the extent of lymph node metastasis and the prognosis, and can tailor post-operative adjuvant chemotherapy for individual patients. For patients with carcinoma of a high proliferative activity, aggressive adjuvant chemotherapy is the policy in our clinics.

We thank M. Ohara for helpful comments. This study was supported in part by a grant-in-aid from Kaibara Morikazu Medical Science Promotion Foundation.

### References

BORCHAD, F. (1990). Classification of gastric carcinoma. *Hepatogastroenterology*, **37**, 223–232.

BORRMANN, R. (1926). Geschwulste des Magens und des Duodenums. In Handbuch Spez Pathol Anat und Histol IV/I, Henke, F. & Lubarsch, O. (eds) pp. 812–1054. Springer: Berlin.

BRAVO, R. & BRAVO, H.M. (1987). Existence of two populations of cyclin/proliferating cell nuclear antigen during the cell cycle: association with DNA replication sites. *J. Cell Biol.*, **105**, 1549–1554.

BRAVO, R., FRANK, R., BLUNDEL, P.A. & BRAVO, H.M. (1987). Cyclin/PCNA is the auxiliary protein of DNA polymerase-delta. *Nature*, **326**, 515–517.

BUSCH, H. (1984). Nucleolar proteins: purification, isolation and functional analysis. In *Chromosomal Non-Histone Proteins*, Hnilica, L.S. (ed.) pp. 233–286. CRC Press: Boca Raton, FL.

COX, D.R. (1972). Regression models and life tables. *J. R. Stat. Soc.* (Series B), **B34**, 187–220.

DIMOVA, R.N., MARCOV, D.V., GAJDARDJIEVA, K.C., DABEVA, M.D. & HADJIOLOV, A.A. (1982). Electron microscopic localization of silver staining NOR-proteins in rat liver nucleoli upon D-galactosamine block of transcription. *Eur. J. Cell Biol.*, **28**, 272–277.

DIXON, W.J. (1988). *BMDP Statistical Software Manual*, pp. 133–744. University of California Press: Berkeley, CA.

EGAN, M.J. & CROCKER, J. (1992). Nucleolar organiser regions in pathology. *Br. J. Cancer*, **65**, 1–7.

FAKAN, S. & HERNANDEZ-VERDAN, D. (1986). The nucleolus and the nucleolar organizer regions. *Biol. Cell.*, **56**, 189–206.

FURUKAWA, H., HIRATSUKA, M. & IWANAGA, T. (1988). A rational technique for surgical operation on Borrmann type 4 gastric carcinoma: left upper abdominal evisceration plus Appleby's method. *Br. J. Surg.*, **75**, 116–119.

IWANAGA, T., FURUKAWA, H., TANIGUCHI, H. & ISHIGURO, S. (1983). Extension modes in Borrmann-4 stomach carcinoma estimated by macroscopic appearance. *Jpn. J. Cancer Clin.*, **29**, 120–124 (in Japanese with English abstract).

JASKULSKI, D., DERIEĽ, J.K., MERCER, W.E., CALABRETTA, B. & BASERGA, R. (1988). Inhibition of cellular proliferation by antisense oligodeoxynucleotides to PCNA/cyclin. *Science*, **240**, 1544–1546.

KAKEJI, Y., KORENAGA, D., TSUJITANI, S., HARAGUCHI, M., MAEHARA, Y. & SUGIMACHI, K. (1991). Predictive value of Ki-67 and argyrophilic nucleolar organizer region staining for lymph node metastasis in gastric cancer. *Cancer Res.*, **51**, 3503–3506.

KAMEI, H., YAMAMURA, Y., ICHIKAWA, K. SUGIMOTO, K., KOJIMA, T., TERABE, K. & KONDE, T. (1981). Mitotic index in gastric carcinoma of the scirrhous type. *Jpn. J. Surg.*, **11**, 337–340.

KURKI, P., LOTZ, M., OGATA, K. & TAN, E. (1987). Proliferating cell nuclear antigen (PCNA/Cyclin) in activated human T lymphocytes. *J. Immunol.*, **138**, 4114–4120.

MAEHARA, Y., MORIGUCHI, S., ORITA, H., KAKEJI, Y., HARAGUCHI, M., KORENAGA, D. & SUGIMACHI, K. (1992). Lower survival rate for patients with gastric carcinoma of Borrmann type IV following gastric resection. *Surg. Gynecol. Obstet.*, **175**, 13–16.

McCORMICK, D. & HALL, P.A. (1992). The complexities of proliferating cell nuclear antigen. *Histopathology*, **21**, 591–594.

MORI, M., KAKEJI, Y., ADACHI, Y., MORIGUCHI, S., MAEHARA, Y., SUGIMACHI, K., JESSUP, J.M., CHEN, L.B. & STEELE, G.D. (1993). the prognostic significance of proliferating cell nuclear antigen (PCNA) in clinical gastric cancer. *Surgery*, **113**, 683–690.

NAGAYO, T. & YOKOYAMA, H. (1974). Scirrhous carcinoma occurring in the corpus (body) of the stomach. *Acta. Pathol. Jpn.*, **24**, 797–814.

NAKAMURA, K., KATO, Y., MISONO, T., SUGANO, H., SUGIYAMA, N., BABA, Y., MURUYAMA, M. & TAKAGI, K. (1980). Growing process to carcinoma of linitis plastica type of the stomach from cancer-development (in Japanese with English summary). *Stomach Intestine*, **15**, 225–234.

PLOTON, D., BOBICHON, H. & ADNET, J.J. (1982). Ultrastructural localization of NOR in nucleoli of human breast cancer tissues using a one-step Ag-NOR staining method. *Biol. Cell.*, **43**, 229–232.

SOWA, M., KATO, Y., NISHIMURA, M., YOSHINO, H., KUBO, T. & UMEYAMA, K. (1989). Clinico-histochemical studies on type 4 carcinoma of the stomach – with special reference to mucopolysaccharides and sialic acid in tumor tissue. *Jpn J. Surg.*, **19**, 153–162.

SUGANO, H., NAKAMURA, K. & KATO, Y. (1982). Pathological studies of human gastric cancer. *Acta Pathol. Jpn.*, **32** (Suppl.), 329–347.

UICC (1987). *TNM Classification of Malignant Tumours*, 4th fully revised edition. Hermanek, P. & Sobin, L.H. (eds) pp. 43–46. Springer: Berlin.

WALKER, R.A. (1988). The histopathological evaluation of nucleolar organizer region proteins. *Histopathology*, **12**, 221–223.

WATSON, J.D., HOPKINS, N.H., ROBERTS, J.W., STEITZ, J.A. & WEINER, A.M. (1987). *Molecular Biology of the Gene*, 4th edn, p. 652. Benjamin/Cummings: Menlo Park, CA.